# Supplementary figures and images for: Genomic Steppe ancestry in skeletons from the Neolithic Single Grave Culture in Denmark
Source: PLoS One. 2021 Jan 14;16(1):e0244872. doi: 10.1371/journal.pone.0244872 (PMC7808695; doi:10.1371/journal.pone.0244872)

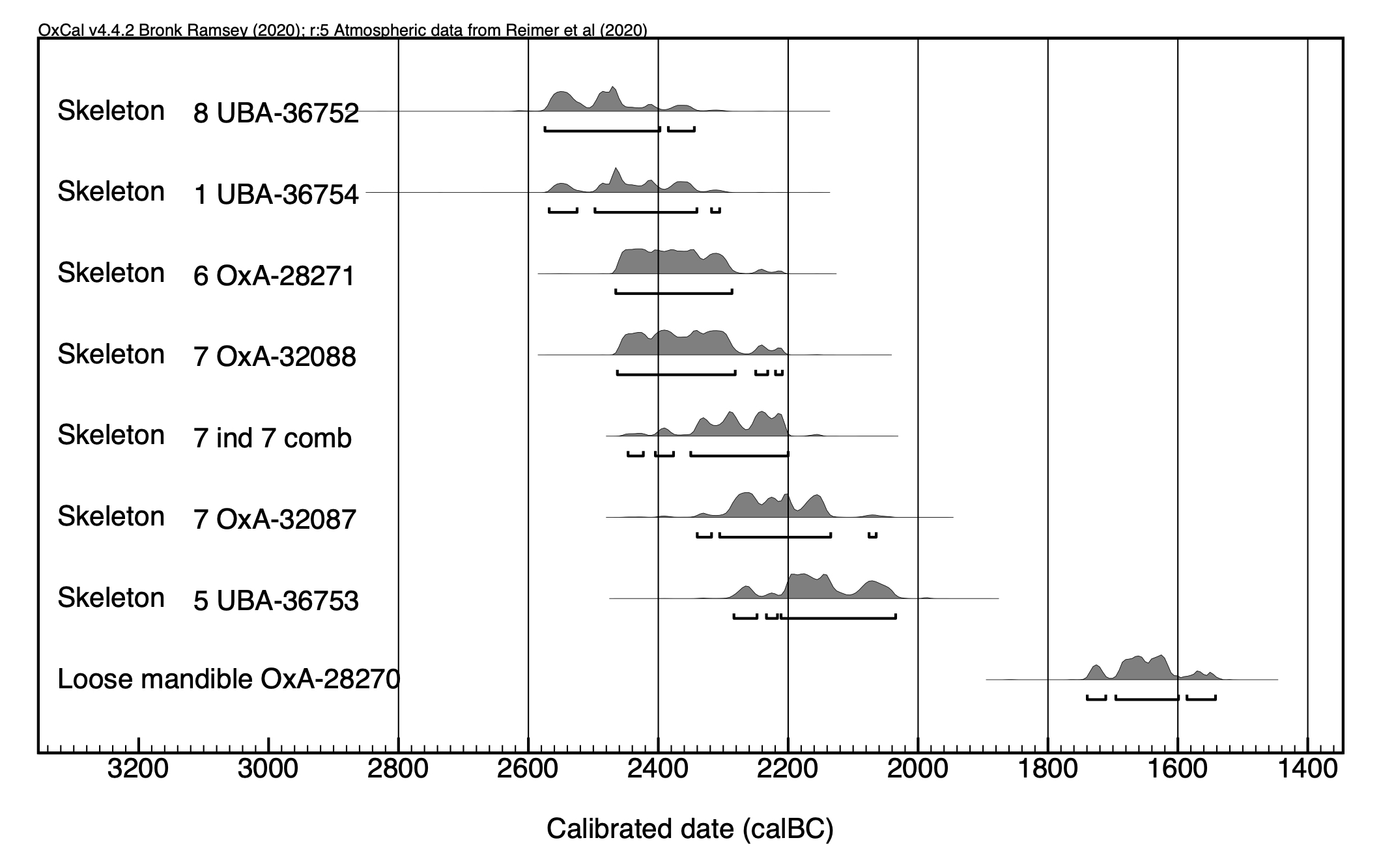

Supplement: S1 Fig — Calibration plot of the radiocarbon dates from Gjerrild, based on reservoir corrected dates. Individual 7 was dated twice as part of a quality control procedure at the Oxford laboratory. A combined date was calculated for this individual. Horizontal bars show 95.4% probability ranges. (TIF) [file pone.0244872.s001.tif]

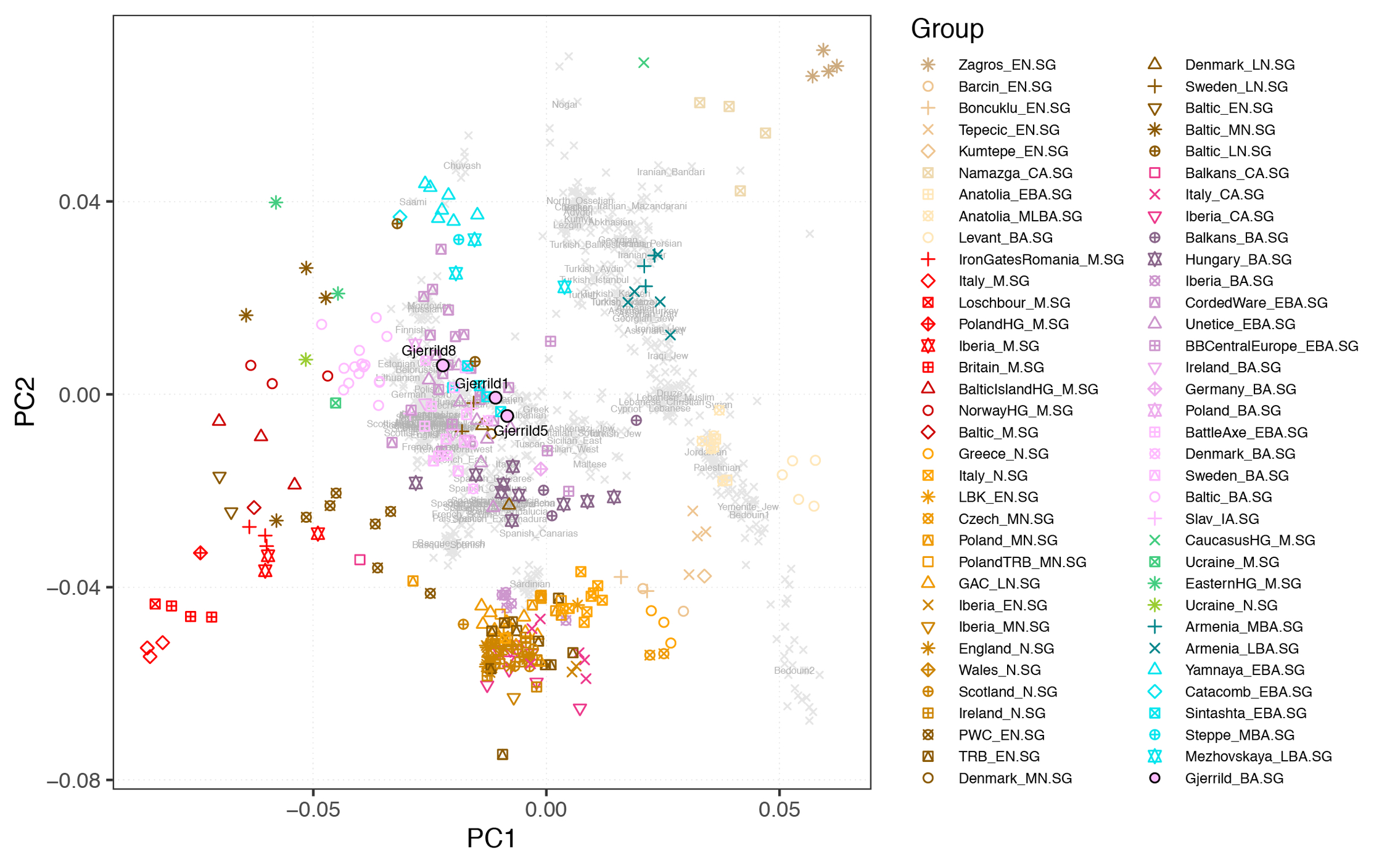

Supplement: S2 Fig — Genetic relationship of the three Gjerrild and other ancient individuals based on PCA. The ancient genomes were projected onto the modern variation of the Affymetrix Human Origins panel. (TIF) [file pone.0244872.s002.tif]
