# Supplementary material for: Genomic Steppe ancestry in skeletons from the Neolithic Single Grave Culture in Denmark
Source: PLoS One. 2021 Jan 14;16(1):e0244872. doi: 10.1371/journal.pone.0244872 (PMC7808695; doi:10.1371/journal.pone.0244872)
Supplement: Attachment — Submitted filename: Gjerrild_ResponseToReviewers_.docx [file pone.0244872.s007.docx]

**Response to comments from editor and reviewers**

We thank the editor and reviewers for their helpful comments, which surely helped to improve the quality of our revised manuscript. We have copy-pasted all comments below. Our responses are in **bold** preceded by ‘>>’.

Your manuscript has now been seen by two referees, whose comments are appended below. You will see from these comments that the referees find your work of great interest, but they have raised several issues that must be fully addressed before re-submission.

Journal Requirements:

When submitting your revision, we need you to address these additional requirements.

1. Please ensure that your manuscript meets PLOS ONE's style requirements, including those for file naming. The PLOS ONE style templates can be found at <https://journals.plos.org/plosone/s/file?id=wjVg/PLOSOne_formatting_sample_main_body.pdf> and <https://journals.plos.org/plosone/s/file?id=ba62/PLOSOne_formatting_sample_title_authors_affiliations.pdf>

**>> We have changed the manuscript accordingly and this updated version should meet the style requirements.**

2. In your Methods section, please provide additional information regarding the permits you obtained for the work. Please ensure you have included the full name of the authority that approved the field or museum site access and, if no permits were required, a brief statement explaining why.

**>> We’ve added the relevant information in the “Materials and Methods” section.**

3. In your manuscript, please provide additional information regarding the specimens used in your study. Ensure that you have reported specimen numbers and complete repository information, including museum name and geographic location. If permits were required, please ensure that you have provided details for all permits that were obtained, including the full name of the issuing authority, and add the following statement:'All necessary permits were obtained for the described study, which complied with all relevant regulations.'If no permits were required, please include the following statement:'No permits were required for the described study, which complied with all relevant regulations.'For more information on PLOS ONE's requirements for paleontology and archaeology research, see <https://journals.plos.org/plosone/s/submission-guidelines#loc-paleontology-and-archaeology-research>.

**>> We’ve added the relevant information in the “Material and Methods” section.**

4. Thank you for stating the following in the Acknowledgments Section of your manuscript: "This research in this study was part of the project “Towards a new European Prehistory” funded by the Riksbanken Jubileumsfond to KK. Centre for GeoGenetics is funded by the Lundbeck Foundation. MEA is supported by the Independent Research Fund Denmark."

We note that you have provided funding information that is not currently declared in your Funding Statement. However, funding information should not appear in the Acknowledgments section or other areas of your manuscript. We will only publish funding information present in the Funding Statement section of the online submission form.

Please remove any funding-related text from the manuscript and let us know how you would like to update your Funding Statement. Currently, your Funding Statement reads as follows:

" KK received funding by the Swedish Riksbanken (The Swedish Foundation for Humanities and Social Sciences) grant M16-0455:1 - Towards a New Prehistory.

<https://www.riksbank.se/en-gb/>

The funders had no role in study design, data collection and analysis, decision to publish, or preparation of the manuscript."

Please include your amended statements within your cover letter; we will change the online submission form on your behalf.

**>> Thanks for noticing this, we have now removed all funding related text from the Acknowledgements section.**

**Please include the following in our Funding Statement "KK received funding by the Swedish Riksbanken (The Swedish Foundation for Humanities and Social Sciences:** [**https://www.riksbank.se/en-gb/**](https://www.riksbank.se/en-gb/)**) grant M16-0455:1 - Towards a New Prehistory. Centre for GeoGenetics is funded by the Lundbeck Foundation. MEA is supported by the Independent Research Fund Denmark. The funders had no role in study design, data collection and analysis, decision to publish, or preparation of the manuscript."**

5. We note that you have stated that you will provide repository information for your data at acceptance. Should your manuscript be accepted for publication, we will hold it until you provide the relevant accession numbers or DOIs necessary to access your data. If you wish to make changes to your Data Availability statement, please describe these changes in your cover letter and we will update your Data Availability statement to reflect the information you provide.

**>> We’ve updated the data availability statement with this sentence: “Sequence data are available at the European Nucleotide Archive under accession number PRJEB40059.”.**

Reviewers' comments:

Reviewer's Responses to Questions

**Comments to the Author**

1. Is the manuscript technically sound, and do the data support the conclusions?

The manuscript must describe a technically sound piece of scientific research with data that supports the conclusions. Experiments must have been conducted rigorously, with appropriate controls, replication, and sample sizes. The conclusions must be drawn appropriately based on the data presented.

Reviewer #1: Yes

Reviewer #2: Yes

2. Has the statistical analysis been performed appropriately and rigorously?

Reviewer #1: I Don't Know

Reviewer #2: Yes

3. Have the authors made all data underlying the findings in their manuscript fully available?

The [PLOS Data policy](http://www.plosone.org/static/policies.action#sharing) requires authors to make all data underlying the findings described in their manuscript fully available without restriction, with rare exception (please refer to the Data Availability Statement in the manuscript PDF file). The data should be provided as part of the manuscript or its supporting information, or deposited to a public repository. For example, in addition to summary statistics, the data points behind means, medians and variance measures should be available. If there are restrictions on publicly sharing data—e.g. participant privacy or use of data from a third party—those must be specified.

Reviewer #1: Yes

Reviewer #2: Yes

4. Is the manuscript presented in an intelligible fashion and written in standard English?

PLOS ONE does not copyedit accepted manuscripts, so the language in submitted articles must be clear, correct, and unambiguous. Any typographical or grammatical errors should be corrected at revision, so please note any specific errors here.

Reviewer #1: Yes

Reviewer #2: Yes

5. Review Comments to the Author

Please use the space provided to explain your answers to the questions above. You may also include additional comments for the author, including concerns about dual publication, research ethics, or publication ethics. (Please upload your review as an attachment if it exceeds 20,000 characters)

**Reviewer #1:**

The paper presents new and significant aDNA analyses for three Neolithic individuals from Jutland and a discussion of their archaeological and genetic contexts. As Jutland is one of the Core Areas of the European Corded Ware, which features key in the current archaeogenetic debates, but is characterized by extremely poor bone preservation, these results are the first of their kind, and thus of high relevance for an international audience and should definitely be published.

It seems to me that the molecular biological investigations (whose details I cannot really assess, due to my lack knowledge). The interpretation of the genetic results in the context of our current archaeogenetic knowledge need some, rather minor, revisions, the way in which genetic and archaeological informations are combined is, in my view at times problematic, however, it is possible to resolve these problems by minor revisions. The archaeological analyses and the final presentation of a migration narrative are in my view a little speculative, but I would argue that they are totally acceptable.

Overall, my recommendation is thus to accept the paper with minor revisions.

Those revisions I would like to recommend are the following:

When it comes to the comparison of archaeological and genetic data there are terminological issues, which really muddy the water, and which should, in my view, be resolved, because they also lead to tautological statements and faulty conclusions. They all derive from the widespread fallacy to treat archaeological cultures as if they would represent specific social groups of humans, and distinct biological populations. This really constitutes a big problem in archaeology. And it is a problem that has plagued the archaeogenetic project from its beginning. It is really time we get past it. The fallacy of treating archaeological cultures like human social groups becomes visible already in the abstract, where it is stated that “

“Assuming that the Gjerrild skeletons are genetically representative of the SGC in broader terms, this culture cannot be regarded as a demographic continuation of the previous Neolithic population.”

**>> We have changed the wording.**

It is more than a pedantic archaeological trivialty: A ‘culture’ in the archaeological sense is a group of archaeological finds, created by archeologists, a population is a unit of biological organisms, defined by biologists. To conflate the two, as it is done in this sentence or to simply equate them has created a lot of misunderstandings. The conflation is the main justification for the assumption at the start: namely that the genetic profiles of three individuals from one single burial could ever be representative for the genetic profiles of all other individuals buried in the context of similar burial types (thus connected to the same archaeological culture). This assumption is a very strong assumption and does, as it stands seem to indicate that all individuals connected to SGC burials would have the same genetic profile. There are actually real, but totally different, arguments to assume that it is in fact correct, but this does not come from the Danish SGC, but rather from the prior knowledge that the great majority (virtually all) individuals connected to Corded Ware Single Graves (a variant of which the SGC burials are) actually have high amounts of steppe ancestry, while all individuals connected to TRB do not. But this cannot be taken fro granted and it does not follow from the analyses of the three Gjerrild individuals alone.

Also “this culture cannot be regarded as a demographic continuation” should be replaced by something like: „the transition from the TRB to SGC is very unlikely to be connected to demographic continuity.”

**>> Addressed!**

This kind of terminology continues on the following pages, where “groups of the Yamnaya culture expand (p. 3)” (I would change that to "groups connected to Yamnaya" – an easy way to get rid of the problematic associations), or the statement that the Pitted Ware Culture (PWC) would have a specific economy (p. 4). Or: “Rather, they genetically resemble individuals from Northern and Eastern Europe that post-date the Yamnaya migrations such as the CWC, Battle Axe Culture and Unetice (p.16).” This can easily be resolved by writing: … such as individuals connected to the CWC, Battle Axe Culture and Unetice.

**>> Addressed!**

That there is more to this than terminological subtleties is illustrated by the fact that it leads exactly to a very simplified research question being supposedly answered, namely a binary "continuity vs. discontinuity of populations":

p. 17: “…and we can therefore reject that SGC in Denmark represents a demographic continuation of earlier Neolithic TRB groups".

**>> Addressed!**

It is unfortunately that the whole discussion is broken down to such a simplistic "continuity or not"- question, which only has any relevance if one presupposes that those units are clear-cut bounded groups of people. This is not to question that migration is obviously an important factor resulting in the shaping of the SGC, but simply to say yes or now to the migration question is really not doing the subject any favour. Especially as later in the archaeological part, continuity of female lineages is basically admitted. So there is a much more interesting story here than the mere identification of “migration as opposed to continuity”. This is then also acknowledged in pages 22-23: “Therefore, when ancient DNA from skeletons of the CWC and related cultures is being analysed, a mix of European Neolithic and Yamnaya-related ancestry is always revealed [1,2].

The ancestry analyses of the Gjerrild skeletons show that the SGC in Denmark could be characterised genetically as a northern ‘branch’ of CWC. As such, the SGC is not a simple demographic continuation of the previous Neolithic TRB people” Why not use this kind of argument already on page 17?

The following sentence (on page 17) is also problematic:

“Rather, our results suggest that the population that lived and buried their dead according to SGC customs should be considered a northwestern branch of the CWC – at least in genetic terms”. This could be reformulated, avoiding the impression that CWC would be something which can be expressed in genetic terms. This is even easier as archaeologically, CWC and SGC are 80-90% identical anyway.

**>> Addressed!**

On page 23 it is stated:

“However, it does seem that the Gjerrild individuals cluster somewhat separate from the main European CWC cluster” This is maybe the place where it also should be acknowledged and discussed that the Gjerrild individuals also do not derive from a typical SGC nor a typical CWC context, which is relevant in this whole discussion. But again I would urge to be careful not to conflate biology and culture.

**>>We have addressed all the above issues and made changes in the text to follow the suggestions.**

Pages 23-24:

“So, if the eastern Swedish PWC individuals can be regarded as genetic representatives of this culture in broader terms, the PWC-SGC interaction occurring on Djursland would be one of two highly distinct population groups, similar to the broader CWC-TRB interaction occurring throughout large parts of Europe at this point in time.

This sentence again illustrates the dangers of the conflation of archaeological cultures and biological populations: a tautology: assuming that individuals connected to Danish PWC would be genetically identical to Swedish PWC (for which there is no real argument), then PWC in Denmark and SGC in Denmark would be different. With other words: assuming they were different, we can conclude they are different…

**>> We address the critique by stating that this proposition needs to be tested. There are material culture arguments for a rather mobile PWC which in all probability shares genetic composition due to their mobility , but this PWC expansion towards northeast Jutland is earlier than our Gjerrild individuals, and corresponds in time to the earlier SGC, which we then discuss in the following section. We have edited the text accordingly.**

P. 24:

“This rare subclade of R1b has previously been observed in three Eneolithic individuals from the Pontic-Caspian Steppe [2,76] but it pre-dates the expansion and diversification in the R1b-M269 lineage, which is typically considered to be associated with the Yamnaya migration and thus common across present-day Europeans. As such the genetic profile of Gjerrild 5 broadens our known Ychromosomal evidence for the links to the early steppe societies.”

This seems like a fact that merits more discussion. Does this mean that the Y-chromosome haplogroup of the Gjerrild individual is not among the ones known from Yamnaya burials? I guess this needs clarification, as the Yamnaya – relation of steppe ancestry is featuring so prominent in the paper. Also, it has become increasingly clear that individuals buried in Yamnaya burials analysed so far are definitely not the biological ancestors of individuals buried in early Corded Ware graves. I would argue that it is in general not really a good idea anymore to narrow down Steppe ancestry to individuals from Yamnaya burials.

**>> In order to show that the Y chromosome lineage of Gjerrild 5 is distinct from those found in Yamnaya-associated individuals, we have expanded the phylogenetic analysis to also include ancient individuals from those contexts, as well as other publicly available individuals carrying R1b-V1636. The new results are shown in updated Figure 3, together with an expanded discussion section on those results.**

And a few really small things:

P.3 “The appearance of the Single Grave Culture (SGC) in Denmark, Northern Germany and Netherlands c. 4850-4600 years ago during the Nordic Middle Neolithic period (MNB)”

They do have different terminologies in Northern Germany (Younger Neolithic), and in the Netherlands (Late Neolithic).

**>> We have changed the wording here and removed the cultural periods.**

P.6 “Hans Christian Petersen carried out a comparative osteological analysis of two Gjerrild craniums that were compared to skulls of the previous TRB, as well as to those of CWC individuals from central Germany [26].” This is a rather uncritical reference to this study. How can something seriously be said to “group with” anything at a sample size of two?

**>> We changed the wording, but none of us are osteologists and we accept the methodological premises in the paper, but we have added that this is indeed a small sample size.**

P. 17 “The SGC covered an area from the Netherlands to Jutland in Denmark where the Gjerrild grave is located.”

The Dutch and the Danish SGCs are not so similar (not more similar at least than Danish SGC and other CWC regions). It is more a coincidence of resaearch history that they ended up having the same name. I would recommend in general to subsume SGC and Battle Axe Culture as variants of CWC, but of course there are different conventions here.

**>> Well different opinions here, so we stay with our formulation.**

Overall, I hope my critical tone in the discussion of individual sentences and statements does not obscure the fact that the study is overall very valuable and has a lot of interesting, relevant insights and arguments. Especially the discussion of migration scenarios into Djursland is an interesting and and productive way of thinking about contextualization of the archaeogenetic findings. Resolving the terminological issues I addressed will, however, make this a much stronger paper. Although I do suggest several revisions, they are all, really, minor.

**>> We appreciate the reviewer’s thorough look at our paper and totally agree that even though there are relatively many things that need to be addressed, all of them are constructive comments/suggestions that will definitely improve the paper. In order to further contextualize the archaeological interpretation of the location of the Gjerrild burial, we have added a little more text on the pottery, and on the flint mines. These contributions come from Poul Otto Nielsen and Lasse Sørensen who we have added to the author list with consent from all other authors. We consider this small text addition to further respond to the reviewers comments**

**Reviewer #2: Broad Comment**

• I really enjoyed this manuscript. Personally, seeing a genetics paper that clearly displays an encyclopedic knowledge of the archaeological context of the finds is both impressive and encouraging. Unfortunately, I feel that some of the findings may be over-ambitious based on the (a) very small sample size, and (b) quality of the samples. I in no way mean this disrespectfully, as finds in this region, and certainly of this age, are rare, and great care and cost was made to produce this data. However, I'd like to see more care taken with the depth of finality to the findings, in light of these limitations.

• I'd really like to see more made of your interpretation of the R1b-V1636 sample in context with other information. The mtDNA HG and the YHG are both Neolithic, and specifically the YHG is rare. However, this individual also carries step ancestry, so how does this compare to the other R1b-V1636 samples? Is this cocktail of history common, to be expected, does it shed any light on the possibility of a "Neolithic ancestry refuge" that was afforded to this population due to their northerly isolation?

**>> See answer to reviewer #1 above, i.e. in order to show that the Y chromosome lineage of Gjerrild 5 is distinct from those found in Yamnaya-associated individuals, we have expanded the phylogenetic analysis to also include ancient individuals from those contexts, as well as other publicly available individuals carrying R1b-V1636. The new results are shown in updated Figure 3, together with an expanded discussion section on those results.**

Page 4:

• “facilitated by migration of people” could be “facilitated by a/the migration of people” or “facilitated by migrations of people”.

**>> Corrected.**

Page 5:

• “well-preserved” seems a little optimistic. Perhaps “relatively well-preserved” better reflects the high quality of the samples, given the typical soil conditions?

**>> Corrected.**

• “With this follows…” could be “From this follows…”.

**>> Corrected.**

Page 6:

• “late in date” is ambiguous. Perhaps “more recent in date”?

**>> Corrected.**

Page 15:

• R1b-V1636 is indeed an interesting haplogroup. Do you state whether it is definitely no more diverged, i.e., ancestral for R1b1a2a and R1b1a2b?

**>> We have added a supplementary table detailing the number of derived SNPs observed in Gjerrild 5 for haplogroup R1b and relevant subhaplogroups, showing that it is ancestral for both R1b1a2a and R1b1a2b.**

• You mention SNP counts for each sample, and percentages, but it might be worth restating that you’re using the 12,731,663 transversions from the 1000GP for clarity.

**>> We have added the total number to the text.**

Page 16:

• Why has PCA not been used here? I’m not suggesting that MDS is not a valid method, but many researchers in this field would likely appreciate a familiar comparison between this research and many other papers by including a classical PCA of West Eurasian modern populations (say that from Haak2015). If the reason is poor coverage on the 1240K SNPs (which I imagine there would be), then this could be stated.

**>> We used MDS on a distance matrix from pairwise identity-by-state counts of the ancient individuals in order to emphasize the genetic structure among the ancient individuals themselves, rather than projecting them on modern genetic variation (see e.g. also Margyaran et al 2020 Nature for a similar approach and discussion). For readers more familiar with the classical projected PCA, we have now also added it as Supplementary Figure S2.**

• Figure 5 the labels have issues in spacing.

**>> We thank the reviewer for noticing, the figure has been updated with a corrected version.**

• “Well-established clustering of the ancient individuals related to age, geography and cultural contexts” is ambiguous for the word age. I assume you mean the calibrated sampling age, and not in fact the age at death of the individual.

**>> Yes, we agree with the reviewer. That part of the sentence now reads “...individuals related to cultural/^14^C date, geography and cultural contexts...”**

Page 17:

• I find the language “and we can therefore reject that SGC in Denmark represents a demographic continuation of earlier Neolithic TRB groups” quite strong language based on one relatively high coverage sample, and two low coverage samples. Specifically, the finding of an individual carrying a rare pre-Steppe R1b Y-haplogroup that also carries Steppe ancestry in the literature, and to assume that this individual (plus two lower-coverage individuals) represents a Denmark-wide culture is perhaps over-reaching slightly based on a small number of (albeit very impressive and rare) samples.

**>>We have changed the wording here.**

Page 22:

• “expanded both eastwards to Altai Mountains and…” should be “expanded both eastwards to the Altai Mountains and…”.

**>> Corrected.**

Page 23:

• Again, using the language “settles a long-lasting debate on the origin of the SGC” is particularly strong, based on the evidence of just three skeletal samples from the same location (even if they come from a 20km-wide radius). I concede that in light of contemporary, and geographically nearby populations (such as northern German), that these findings likely will stand up to (hopefully) further finds, however, I still find the finality of the language a little too strong. The authors do state that due to the limitations of the sample numbers and quality, that finding the exact origin of the Danish SGC is unrealistic, although it’s actually likely impossible at this stage. I’d like to see this reservation extended to other facets of the interpretations.

**>> We agree with the review and have changed the text accordingly.**

Page 24:

• Another Eneolithic R1b-V1636 individual was also found in Arlanstepe, Turkey [Skourtanioti2020].

**>> We thank the reviewer for pointing this out, we have now added this in the text.**
